# Bayesian Latent Class Models in Malaria Diagnosis

**DOI:** 10.1371/journal.pone.0040633

**Published:** 2012-07-23

**Authors:** Luzia Gonçalves, Ana Subtil, M. Rosário de Oliveira, Virgílio do Rosário, Pei-Wen Lee, Men-Fang Shaio

**Affiliations:** 1 CEAUL and Unidade de Saúde Pública Internacional e Bioestatística, Instituto de Higiene e Medicina Tropical, Universidade Nova de Lisboa, Lisboa, Portugal; 2 CEMAT and Departamento de Matemática, Instituto Superior Técnico, Universidade Técnica de Lisboa, Lisboa, Portugal; 3 CMDT/LA and Unidade de Parasitologia Médica, Instituto de Higiene e Medicina Tropical, Universidade Nova de Lisboa, Lisboa, Portugal; 4 Institute of Biomedical Nutrition, HungKuang University, Taichung, Taiwan; 5 Department of Tropical Medicine, National Yang-Ming University, Taipei, Taiwan; Swiss Tropical & Public Health Institute, Switzerland

## Abstract

**Aims:**

The main focus of this study is to illustrate the importance of the statistical analysis in the evaluation of the accuracy of malaria diagnostic tests, without admitting a reference test, exploring a dataset (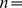
3317) collected in São Tomé and Príncipe.

**Methods:**

Bayesian Latent Class Models (without and with constraints) are used to estimate the malaria infection prevalence, together with sensitivities, specificities, and predictive values of three diagnostic tests (RDT, Microscopy and PCR), in four subpopulations simultaneously based on a stratified analysis by age groups (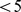, 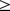 5 years old) and fever status (febrile, afebrile).

**Results:**

In the afebrile individuals with at least five years old, the posterior mean of the malaria infection prevalence is 3.2% with a highest posterior density interval of [2.3–4.1]. The other three subpopulations (febrile 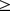 5 years, afebrile or febrile children less than 5 years) present a higher prevalence around 10.3% [8.8–11.7]. In afebrile children under-five years old, the sensitivity of microscopy is 50.5% [37.7–63.2]. In children under-five, the estimated sensitivities/specificities of RDT are 95.4% [90.3–99.5]/93.8% [91.6–96.0] – afebrile – and 94.1% [87.5–99.4]/97.5% [95.5–99.3] – febrile. In individuals with at least five years old are 96.0% [91.5–99.7]/98.7% [98.1–99.2] – afebrile – and 97.9% [95.3–99.8]/97.7% [96.6–98.6] – febrile. The PCR yields the most reliable results in four subpopulations.

**Conclusions:**

The utility of this RDT in the field seems to be relevant. However, in all subpopulations, data provide enough evidence to suggest caution with the positive predictive values of the RDT. Microscopy has poor sensitivity compared to the other tests, particularly, in the afebrile children less than 5 years. This type of findings reveals the danger of statistical analysis based on microscopy as a reference test. Bayesian Latent Class Models provide a powerful tool to evaluate malaria diagnostic tests, taking into account different groups of interest.

## Introduction

Malaria is caused by *Plasmodium* parasites that infect humans through the bites of an infected female mosquito of the genus *Anopheles*. *Plasmodium falciparum*, *P. vivax*, *P. ovale* and *P. malariae* are the main species of malaria parasites. The first two species cause the most infections worldwide [1]. The World Malaria Report 2010 [2] summarizes information from 106 malaria-endemic countries (and 2 countries that were certified as free of malaria in 2010: Morocco and Turkmenistan). This report estimated that the number of cases of malaria changed from 233 million in 2000 to 225 million in 2009. The number of deaths due to malaria is estimated to have decreased from 985 000 in 2000 to 781 000 in 2009. As pointed out by Wongsrichanalai et al. [3], the discrepancy found in worldwide malaria statistics (values range from 300 to 500 millions cases a year) emphasizes the importance of correctly diagnosing malaria to better understand its true extent.

The good clinical practice recommends the parasitological confirmation of the diagnosis of malaria through microscopy. There are some exceptions, namely for children under the age of 5 years in high prevalence areas, where there is no evidence that the benefits of microscopy confirmation exceed the risk of not treating false negatives, for cases of fever in established malaria epidemics where resources are limited and for locations where good quality microscopy is not feasible [1]. This method is cheap, but time-consuming, labor intensive and depends on the quality of the blood films and the expertise of the lab technicians.

In recent years, a variety of rapid diagnostic tests (RDTs) have been explored (e.g. [4]–[8]). RDTs are often more costly than microscopy and this should be borne in mind when deciding purchase quantities and level of use in a health care system [1]. Rapid diagnostic tests may have a crucial role in malaria control in poor countries [3]. On the other hand, even in the United States, according to Stauffer et al. [9], approximately 4 million travelers to developing countries seek health care, with 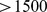 cases of malaria reported annually. These authors explored the performance of a RDT approved by the US Food and Drug Administration, pointing out that the diagnosis of malaria is frequently delayed by physicians who have no tropical medicine experience and by lack of the technical expertise.

Molecular techniques such as polymerase chain reaction (PCR) and quantitative nucleic acid sequence bases amplification are also used, but are not widely used in resource-limited settings [10].

In this work, a statistical analysis will be carried out to explore the performance of three diagnostic tests – a Rapid Diagnostic Test (RDT), the Microscopy and a Polymerase Chain Reaction (PCR) technique – applied in 3317 blood samples collected in São Tomé and Príncipe. In 2005, this country began an initiative aimed at reducing malaria-related mortality to zero [11]. Lee et al. [12], [13] present some results on pre-elimination of malaria on the island of Príncipe and show a remarkable decline in malaria morbidity and mortality after the implementation of an integrated malaria control programme in 2004. According to World Malaria Report 2010 [2], São Tomé and Príncipe belongs to a group of 11 African countries that showed a reduction of more than 50% in either confirmed malaria cases or malaria admissions and deaths in recent years due to intense malaria control interventions. However, the World Malaria Report 2010 [2] points out that in 2009 there was evidence of an increase in malaria cases in São Tomé and Príncipe. This report notes that *“the increases in malaria cases highlight the fragility of malaria control and the need to maintain control programmes even if numbers of cases have been reduced substantially”*.

Statistical analysis is crucial to validate diagnostic tests. The development of classic (frequentist) and Bayesian statistical approaches for evaluation of the diagnostic tests in the absence of a gold standard test has been an active field of biostatistical research applied to many areas, including tropical diseases (e.g. [14], [15]), oncology [16], [17] and veterinary medicine [18]. Latent class models with two latent classes are widely used to estimate the prevalence, sensitivities and specificities in the absence of a gold standard. Microscopy has been considered as the gold standard for malaria diagnosis. However, admitting microscopy as a reference technique impairs the sensitivity and specificity estimation for other diagnostic techniques [19]. Bayesian approaches are increasingly being used in the analysis of parasitological data, including in the performance of diagnostic tests. Menten el al [20] present several Bayesian latent class models for the diagnosis of visceral leishmaniasis. Limmathurotsakul et al. [21] explore some diagnostic tests for melioidosis. In malaria, as the best of our knowledge, few papers explore latent class models. Speybroeck et al. [14] present a contribution of a Bayesian approach to estimate the prevalence of malaria, applying ELISA, PCR and microscopy to datasets from Peru, Vietnam, and Cambodia. Ochola et al. [19] use a Bayesian formulation of the latent class model of Hui and Walter to estimate the diagnostic accuracy of the malaria diagnostic techniques and microscopy in the absence of a gold standard, based on a systematic review. Fontela et al. [22] point out the poor methodological quality and/or poor reporting of published diagnostic accuracy studies on commercial tests for the three major infections: tuberculosis, malaria and human immunodeficiency virus. In this work, our goal is to explore the accuracy of three diagnostic tests for malaria, using Bayesian Latent Class Models (BLCM), considering their performances in four populations based on the combination of age groups (less than 5 years, greater than or equal to 5 years) and fever status (febrile, afebrile). BLCM without and with restrictions (also called constraints) are used to estimate the disease prevalence, together with sensitivities, specificities, and predictive values of the diagnostic tests. The choice of this type of models will be discussed in the next sections.

## Materials and Methods

### Malaria Diagnosis Data

In São Tomé and Príncipe (STP), a malaria programme was officially initiated in 2004, and a molecular diagnostic laboratory was set in the main island of São Tomé in 2007, following STP government directives for malaria control and for ethical clearance throughout the implementation of the programme. In the context of this program, between July 2008 and August 2009, a household survey provided data on three mentioned above diagnostic tests applied in 3317 blood samples. The households were selected randomly. Ethical approval was obtained from the Ministry of Health of the Democratic Republic of STP. Informed verbal consent was obtained from residents who answered a short questionnaire, which included information on the use of bed nets. Parents responded on behalf of infants and children [13]. It was expected and observed that the participants in the research are illiterate or semi-literate, therefore could not sign a written consent. This study took into account that the principles of verbal informed consent were the same for written informed consent. This procedure was approved by Ministry of Health of the STP. The body temperature (ear) was also collected and recodified into fever status (Febrile and Afebrile) taking into account a cut-off of 37.5°C for fever. The age groups were defined according to WHO recommendations - 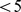 and 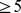 years old - considering the importance of under-five children that are mainly affected by anaemia and mortality [23].

The central statistical analysis of the three diagnostic tests –1. RDT, 2. Microscopy and 3. PCR – (binary variables taking the values: 1. positive *versus* 0. negative) will take into account a unique dataset of the four subsamples defined by the combination of age groups and fever status, as indicated in Table 1, and it will be presented in the next section.

**Table 1:** Subsamples defined by the combination of age groups and fever status and observed frequencies of each pattern of test results.

Tests	Afebrile	Febrile	Afebrile	Febrile
(1,2,3)	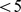 years	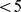 years	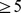 years	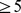 years
1 1 1	20	22	40	98
1 1 0	1	1	0	0
1 0 1	24	2	9	2
1 0 0	28	4	19	19
0 1 1	1	1	1	1
0 1 0	1	0	3	1
0 0 1	1	1	2	1
0 0 0	419	210	1535	850
Total	495	241	1609	972

All cases were tested by rapid diagnostic tests (RDTs, ICT Diagnostics, Cape Town, South Africa), with blood films prepared for microscopic examination, and with dry blood spots collected on filter papers (FTA Classic Cards, Whatman, Newton, MA) for PCR as previously reported [24], [25]. Two technicians for each of six teams carried out the RDT together and decided on the reading. Three technicians recording the microscopic result were unaware of the corresponding RDT results. The technicians that performed the microscopic examination and PCR did not know the age groups and the fever status of the patients. However, the technicians that applied the RDT knew the age groups and the fever status of the individuals because they also made the demographic record of all cases including sex, age, body weight, and temperature.

### Some Points regarding the Statistical Analysis

#### Confidence intervals in the classical analysis - using a reference test

It is still common in medical literature, the classical statistical approach which admits the microscopy as a gold standard. This approach has been criticized also in a malaria context [14], [19]. An important related issue is also the confidence intervals that accompany the point estimate for the sensitivity or the specificity (or other proportions). This problem is not much addressed in medical literature but is still present even when a true gold standard is considered. Usually a 95% confidence interval (95% CI) is obtained by the Wald method that has been strongly criticized due to the poor coverage probability, even for large sample sizes, and the possibility of lower and upper limits outside 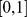 [26]–[28]. To avoid the latter drawback, we recommend the version of the Wald CI, and other methods, given by Pires and Amado [26]. However, as poor coverage probability remains, other alternative methods for constructing confidence intervals should be used. There are a lot of alternative methods that are re-emerging, for example, the Clopper-Pearson (or exact binomial), Wilson (or score), Agresti-Coull and Jeffreys methods that provide more reliable coverage probabilities than the Wald method. In the context of diagnostic tests, Wilson method was recommended by [29]. In risk situation, when a coverage probability must be guaranteed, a conservative method (e.g. Clopper-Pearson) may present advantages. Nevertheless, these and other recommended methods may also present coverage problems near the boundaries (0 or 1) [26]–[28]. Some of these are available from R Packages or Epitools [30] (caution should be taken regarding the problem of limits outside 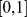). The key to avoid troubles is to use several recommended methods to understand if they provide consistent information. The mathematical expressions of the methods used in this work can be found in Table 2.

**Table 2:** Lower – 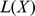 – and upper – 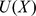 – bounds of a 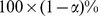 confidence level for a two-sided confidence interval – 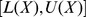 – for a proportion 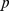 (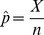, where 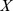 is the number of successes) using different methods.

Method	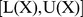
Wald	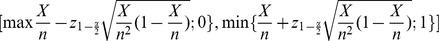
Clopper-	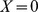 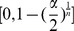 , if
-Pearson	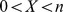 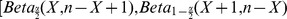 ], if
	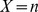 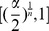 , if
Wilson	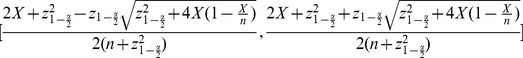
Agresti-	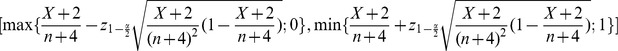
-Coull	
Jeffreys	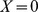 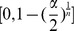 , if
	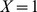 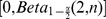 , if
	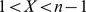 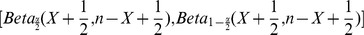 , if
	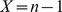 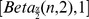 , if
	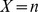 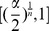 , if

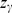
 and 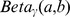 represent the 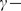
quantiles of the 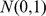 and the 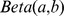 distributions, respectively.

#### Latent class models

Ignoring the limited precision of a reference test can incur serious bias in the performance of other medical diagnostic tests and also in the prevalence estimation. Frequentist and Bayesian latent class models are important mathematical frameworks to study the prevalence and the performance of diagnostic tests in the absence of a gold standard test. In a Bayesian analysis, data are combined with the prior information that expresses expert opinions and other sources of knowledge. The elicitation of an informative prior is a hard and subjective process that needs a careful dialogue between statisticians and experts. Despite the existence of a broad and diverse literature in elicitation of prior distributions, it is mainly oriented to statisticians and not to experts in other fields. However, a vast literature has emphasized the importance of prior information. Speybroeck et al. [14] and the references therein point out the merits of the Bayesian paradigm in the estimation of the parameters associated with three diagnostic tests and the prevalence of malaria infection.

In a frequentist perspective, the parameters of latent class models can be obtained by the well-known Expectation Maximization (EM) algorithm. In a Bayesian approach, the parameters are usually estimated by Markov Chain Monte Carlo (MCMC) methods, via Gibbs sampling. The simplest model is the Two Latent Class Model (2 LCM). In this model, the true disease/infection status of an individual is considered a latent variable, 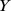, with two mutually exclusive categories (1. diseased/infected and 0. non-diseased/non-infected). The manifest binary variables, 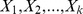, that express the 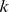 diagnostic tests results, only give an indication on disease/infection status. The 2 LCM assumes that, given the true state of the disease or infection, the results of the diagnostic tests are independent. This assumption is known as Hypothesis of Conditional Independence (HCI) and it will be discussed in the next subsection. Frequentist and Bayesian latent class models, and their extensions to more complex settings, require a careful analysis of several points to ensure reliable results.

#### Hypothesis of conditional independence

According to the parsimony principle, mathematical models with the smallest number of parameters are preferred to the more complex ones. However, to investigate if the simplest and most parsimonious 2 LCM describes the data adequately, we need to check if the HCI is or not violated. The HCI in some medical problems may not be a realistic assumption, for example, when the two tests are based on a similar biological phenomenon (e.g. [20], [31]). The diagnostic of local dependence has been discussed by several authors [31]–[35] and different methods have been proposed. Among others, Hagenaars [32] suggests the analysis of the standardized residuals for each pair of manifest variables. Garrett and Zeger [34] developed a graphical method, the log odds ratio check (LORC) plot, to compare the log odds ratio for the observed and predicted two-way cross classification tables for each pair of manifest variables. Qu et al. [35] also propose a graphical method, the correlation residual plot, which is obtained by plotting residuals of pairwise correlation coefficients, defined as the difference between the observed and expected correlations. Sepúlveda et al. [31] propose the use of Biplot representations based on generalized linear models to identify conditional dependence between pairs of manifest variables within each latent class. In this field, Subtil el al. [36] simulated data incorporating local dependence between pairs of manifest variables and applied different local dependency diagnostic methods and found some problems in the detection of the violation of the principle of conditional independence. In case of failure of HCI, there are alternative approaches to 2 LCM. Alternative models that accommodate conditional dependencies have been proposed in the last decade. Albert and Dodd [37] present an overview of some modeling approaches to incorporate conditional dependence between tests. Qu et al. [35] and Hadgu and Qu [38] developed a general latent class model with random effects to incorporate possible conditional dependencies among diagnostic tests. Additionally, Dendukuri and co-authors presented models in a Bayesian perspective [39], [40]. The accessibility of MCMC methods provide solutions to complex models in evaluation of diagnostic tests [41]. On the other hand, the knowledge transfer from other areas may also contribute to this medical field. In particular, sociology and psychology offer solid methodological developments in the latent class models that may be useful in the context of multiple diagnostic tests, as pointed out by Formann [42].

#### Non-identifiability and label-switching problem

The non-identifiability of latent class models is a sensitive issue that requires careful attention. If models are not identified, there will not be a unique computational solution. Jones et al. [43] and the references therein give an overview on identifiability of models for multiple binary diagnostic tests in the absence of a gold standard. Apart from checking trivial conditions, such as that the number of parameters has to be smaller than the number of different patterns, in general, for complex models it is not possible to say a priori whether a model is or not identifiable [42].

An advantage of the Bayesian approach is the incorporation of the prior information to avoid the non-identifiability. When the model is identifiable, non-informative prior distributions can be used for all parameters [40]. When the model is not identifiable, it may still be possible to obtain a solution, adding constraints on the parameters or/and by using informative prior distributions for some parameters (e.g. [44] and [40] and the references therein). In practice, a special attention should be given to the non-identifiability under symmetric priors that leads to the label switching in the MCMC output [45] produced in the parameter estimation process. Label switching occurs when latent classes change meaning over the estimation chain in the context of MCMC. Other types of estimation (e.g., maximum likelihood estimation) can exhibit this problem [46]. Machado et al. [47] show the graphical behavior of the traceplots and posterior densities for the latent class probabilities with a label-switching problem. Stephens [48] points out that the common strategy of removing label switching by imposing artificial identifiable constraints on the model parameters does not always provide a satisfactory solution. In fact, there is an active scientific debate in many fields and other solutions have been proposed in the literature (*e.g.* [45], [48]–[51]). On the other hand, for the situations in which two diagnostic tests are applied in two populations (the Hui-Walter paradigm), Gustafson [52], [53] demystifies the conventional view of identifiability – “*identifiability good, non-identifiability bad*”–, presenting realistic scenarios where a moderate amount of prior information leads to reasonable inferences from a non-identified model, and scenarios where large sample sizes may be required to obtain reasonable inferences from an identified model.

#### Sampling strategies or stratified analysis

The product-multinomial model appears naturally when we collect independent samples on a number of subpopulations corresponding to the traditional stratified sampling [54]. Gustafson [52] explores one way to develop an identifiable model through pre- or post-stratification of the sample/population according to some categorical variable. Dohoo [55] and Gardner et al. [56] argue that it is acceptable to artificially construct populations with a practical meaning. The post-stratification is a way to overcome some situations, where it is inconvenient or impossible to stratify a population into strata before sampling because the value of the variable of interest is only observed after the individual is sampled. In our application, described before, the variable fever could be an example of this type.

In medical problems, the relevance of distinguishing between subsets is very important to understand if the performance of a diagnostic test varies across smaller groups. As an example, the World Malaria Report 2010 [5] emphasizes that “*the clinical sensitivity of an RDT to detect malaria is highly dependent on the local conditions, including parasite density in the target population, and so will vary between populations with differing levels of transmission*”. In order to estimate the prevalence and the performance measures of several diagnostic tests in the absence of a gold standard, in two or more distinct populations, BLCM are widely used by the veterinary community [57], [58], where subpopulations (e.g. herds) appear naturally or are created (e.g. [59]). On the other hand, Martinez et al. [16] present a Bayesian approach to estimate the disease prevalence, and the accuracy of three screening tests in the presence of two covariates (age, pregnancy) in the absence of a gold standard for cervical cancer. A logit link function was used to relate the covariates linearly to the screening performance measures to provide a meaningful and well-known measure of association - odds ratio. Posterior odds ratios as association measures between pregnancy and age and the performance measures of the three tests and prevalence are presented. This approach is of great importance in the discovery of potential effects of covariates in the sensitivity, specificity and prevalence. If these effects are already known, it seems to be appropriate to choose a stratified analysis, providing the performance measures of each test in each stratum. In a first view the study design - a random survey - seems to suggest a Latent Class Model with covariates [60], however, as described before, the technicians that applied the RDT knew the age groups and the fever status of the individuals. Thus, the stratified analysis is the chosen approach to the malaria dataset.

### Bayesian Latent Class Models without and with Constraints

In biomedical sciences, data from multiple dichotomous diagnostic tests arise from multinomial or product-multinomial distributions depending upon the number of populations [43]. The well-known Hui-Walter model involves a split of the population into two or more populations – (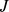)– and assuming conditional independence of the 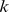 tests given the disease status; the sensitivity and specificity should be constant across populations and the prevalence of the disease is different within each population. This model becomes identifiable whenever 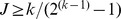 (see [19], [61], [62]).

In this work, the subsamples 1, 2, 3 and 4 are drawn from subpopulations 1, 2, 3, and 4, where the corresponding malaria infection prevalence is denoted by 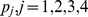. The sensitivity of test 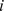 (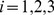) in the subpopulation 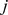 (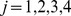) is denoted by 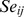. Similarly, 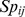 represents the specificity of test 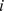 in the subpopulation 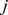. We continue to assume conditional independence of the 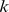 tests given the disease status, however, the prevalence, the sensitivities, and the specificities may vary across subpopulations. For cancer ascertainment data, Bernatsky et al. [17] considered this situation but used a latent class hierarchical model. Here, we adopt a different approach, considering constraints on the general model to obtain other simpler models to model our data set.

In this work, we admit that the 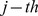 subpopulation counts (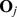) of the different patterns of test results (in a total of 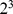 possible patterns) follow a multinomial distribution:


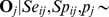
 Multinomial 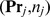, 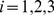 and 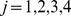,

where 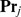 is a vector of probabilities of observing the individual pattern 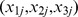 of test results in population 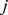 (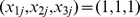 to 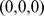 as shown in the first column of Table 1) giving by.
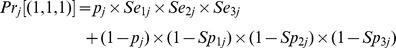


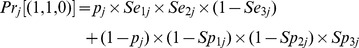


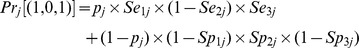


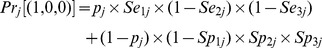


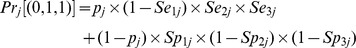


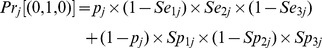


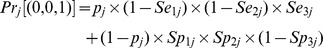


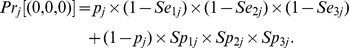



To analyze the four subpopulations simultaneously, a product multinomial distribution is considered simply using the product of four multinomial distributions since the subpopulations are independent. This general model may be simplified to obtain other simpler models, using constraints. For example, the notation 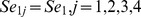 means that RDT test presents the same sensitivity across the four subpopulations of interest. In a general way, 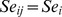 and 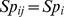 means that the sensitivity and specificity of the test 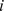 are constant over subpopulations. This simplest model (denoted by M1 in next section) with constraints considers a different prevalence for each subpopulation and the sensitivities and specificities of each test are the same across subpopulations. This model is commonly used to evaluate diagnostic tests in two or more populations (see [19], [58], [61]). The general model (no constraints are imposed on prevalence, sensitivities and specificities across subpopulations, M2 in the next section) has 28 parameters and the simplest model has only 10 parameters to be estimated, using a Bayesian approach. Introducing different constraints into M2, several other Bayesian latent class models were fitted via MCMC techniques, using Gibbs sampling, to explore the accuracy of the three diagnostic tests in the four defined subpopulations (Table 1) simultaneously.

Berkvens et al. [44] consider two types of constraints - deterministic and probabilistic. Both types of constraints express previous knowledge on parameters of a model and/or are imposed to overcome the non-identifiability of a model. The last one appears in a Bayesian context to reflect the available knowledge and uncertainty, specifying a prior distribution for a parameter. Informative priors are based on historical information, expert opinions, beliefs based on the repetition of similar experiments, and so on. If previous information is not available, a non-informative or a vague prior distributions are commonly used.

The elicitation of an informative prior is a hard and subjective process that needs a careful dialogue with experts. Despite the existence of a broad and diverse literature in elicitation of prior distributions, it is mainly oriented to statisticians and not to experts in other fields. In practice, user-friendly graphical tools are essential to lead with this sensitive issue. In this process, we used Epitools [30] to summarize Beta distributions for specified 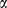 and 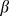 parameters – Beta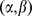. In our opinion, the flexibility of Beta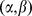 seems to be more natural than the Uniform distributions to describe probabilistically this type of performance parameters. We should note that an Uniform over the interval [0,1] is equivalent to a Beta(1,1). If previous studies have been pointed out that a particular test presents a sensitivity most of times concentrated near 1, choosing a right-skewed beta distribution with parameters 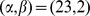, with a standard deviation of 0.053 and theoretical quantiles 0.025, 0.50 and 0.975 equal to 0.789, 0.931 and 0.990, expresses a better performance than the another right-skewed Beta distribution with parameters 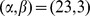 (standard deviation of 0.062 and quantiles: 0.025, 0.50 and 0.975 equal to 0.740, 0.895 and 0.975). A left-skewed distribution suggests a trend to a poor performance of a test or a low prevalence. For example, according to an expert, the probability of malaria infection prevalence lower than 0.15 is equal to 0.95. Additionally, he/she considers that the mean, mode and median are approximately 0.10. A Beta(15,131) seems to be a good candidate to express this information.

Some computer programming to evaluate the BLCMs was implemented in WinBUGS 1.4.3 program [63]. Appendix S1 shows an example of the code corresponding to model M5. The R statistical software version 2.80 [64] was also used to benefit from the package R2WinBUGS. In general, inferences were based on 100,000 iterations after discarding an initial burn-in of 5,000 iterations with convergence assessed by running multiple chains from various starting values [65]. All parameters were estimated with 95% credible intervals (Bayesian version of the confidence intervals). Additionally, the highest probability density (HPD) intervals for parameters of interest were obtained using BOA 1.1 7–2 [66]. These results will be presented later. Convergence was monitored using the standard diagnostic procedures based on a visual assessment of the long chains for each parameter and using the Gelman-Rubin and the Raftery-Lewis measures. The first requires a 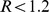 and the last one a dependence factor 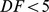 [66].

In terms of model selection, the Deviance Information Criterion (DIC) [67] which penalizes goodness of fit by “complexity” (with the last one measured by effective number of parameters) was valued. The model with the smallest DIC should be selected. However, if two competing models differ in DIC by less than three units, the models are not considered statistically different [62], [67]. The BUGS project [68] gives some guidelines suggesting that differences of more than 10 might definitely rule out the model with the higher DIC, differences between 5 and 10 are substantial, but if the difference in DIC is less than 5, and the models produce very different inferences, then it could be misleading just to report the model with the lowest DIC. This criterion is a generalization of the Akaike Information Criterion (AIC) and Bayesian Information Criterion (BIC) that are also presented (Tables 3 and 7). In models with negligible prior information, DIC will be approximately equivalent to AIC [67]. Note that we use these measures as a comparison criteria to select a model from a set of two-latent class models fitted to a particular dataset. In the literature on latent class models, some criticism has been reported when DIC, AIC and BIC are used to choose the number of latent classes [69], [70] and some variants have been proposed. To assess the adequacy of the selected model, the Bayesian *p*-value [71], based on Pearson statistics, was also calculated as described in detail by Nérette et al. [62]. This version of Bayesian *p*-value suggests the lack of fit when *p*-values near 0 or 1 [62], [72]. Other versions and interpretations of Bayesian *p*-value can be found, as well in the context of latent class models [44]. There is some subjectivity in the choice of a cut-off to indicate the adequacy of a model, as pointed out by Neelon et al. [72], by analogy to the frequentist *p*-value, a Bayesian *p*-value in (0.05, 0.95) suggests an adequate fit, although, in some cases, a stricter criterion might be more appropriate and the values should be in (0.20, 0.80). Ideally, the *p*-value should be close to 0.5 to express an adequate model fit [20], [72], [73].

**Table 3:** Some measures for the selected models (under HCI).

Measures	M1	M2	M3	M4	M5
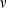	10	28	22	26	20
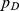	9.12	16.24	16.20	16.19	14.30
AIC	207.72	170.01	152.02	164.28	146.32
BIC	268.79	341.00	286.37	323.06	268.45
DIC	196.84	130.26	124.22	128.47	120.62
Bayesianp-value	0.000	0.477	0.638	0.552	0.703

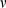
 - Number of parameters to be estimated.

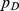
 - Effective number of estimated parameters.

**Table 4:** Bayesian estimates of prevalence, sensitivities, specificities and predictive values, given by posterior means and 95% credibility intervals – Mean [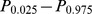] – with non-informative priors, by age groups and fever status, using the models M3, M4 and M5.

Parameters	M3	M4	M5
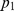	10.1 [7.5–13.2]	10.4 [8.9–11.9]	10.4 [9.0–12.0]
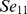	94.3 [85.8–99.1]	94.7 [86.4–99.3]	94.3 [85.7–99.1]
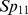	94.0 [91.5–96.4]	94.0 [91.5–96.3]	94.1 [91.5–96.3]
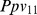	64.0 [51.6–77.1]	64.9 [54.9–75.5]	65.1 [55.0–75.6]
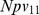	99.3 [98.2–99.9]	99.3 [98.4–99.9]	99.3 [98.2–99.9]
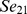	45.2 [31.4–59.4]	45.3 [31.4–59.6]	45.0 [31.2–59.2]
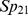	99.8 [99.6–99.9]	99.6 [98.7–100.0]	99.8 [99.6–99.9]
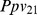	96.3 [92.1–98.8]	92.2 [79.2–99.3]	96.4 [92.6–98.8]
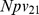	94.2 [91.6–96.2]	94.0 [92.2–95.7]	94.0 [92.2–95.6]
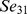	91.2 [76.7–99.1]	91.4 [77.4–99.2]	91.1 [77.2–99.0]
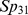	99.9 [99.7–100.0]	99.6 [98.8–100.0]	99.9 [99.7–100.0]
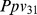	98.5 [96.6–99.7]	96.8 [89.8–99.9]	98.6 [96.9–99.7]
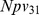	99.0 [97.0–99.9]	99.0 [97.4–99.9]	99.0 [97.3–99.9]
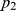	11.4 [7.7–15.7]	10.4 [8.9–11.9]	10.4 [9.0–12.0]
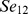	91.4 [78.4–98.8]	92.4 [79.9–99.0]	91.5 [78.5–98.8]
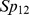	97.8 [95.4–99.4]	97.8 [95.3–99.4]	97.8 [95.4–99.4]
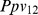	84.3 [69.1–95.3]	83.0 [68.9–94.4]	83.1 [69.0–94.2]
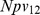	98.9 [97.0–99.8]	99.1 [97.6–99.9]	99.0 [97.5–99.9]
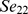	87.1 [72.1–97.1]	88.2 [73.2–97.6]	87.3 [72.3–97.1]
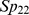	99.8 [99.6–99.9]	99.5 [98.2–100.0]	99.8 [99.6–99.9]
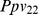	98.3 [96.3–99.4]	95.6 [84.9–99.9]	98.1 [96.3–99.4]
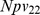	98.4 [96.2–99.6]	98.7 [96.9–99.7]	98.5 [96.8–99.7]
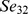	92.1 [79.0–99.0]	92.3 [79.4–99.1]	92.2 [79.3–99.1]
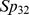	99.9 [99.7–100.0]	99.1 [97.4–99.9]	99.9 [99.7–100.0]
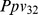	98.7 [96.9–99.8]	92.6 [80.2–99.4]	98.6 [96.9–99.7]
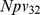	99.0 [97.2–99.9]	99.1 [97.6–99.9]	99.1 [97.6–99.9]
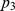	3.2 [2.4–4.1]	3.2 [2.4–4.1]	3.2 [2.4–4.1]
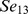	95.3 [87.5–99.4]	95.3 [87.5–99.4]	95.3 [87.8–99.4]
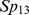	98.7 [98.1–99.2]	98.7 [98.1–99.2]	98.7 [98.1–99.2]
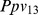	71.4 [60.2–81.4]	71.4 [60.1–81.5]	71.4 [60.0–81.5]
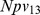	99.8 [99.6–100.0]	99.8 [99.6–100.0]	99.8 [99.6–100.0]
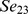	79.9 [67.9–89.6]	79.7 [67.6–89.6]	79.8 [67.7–89.7]
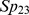	99.8 [99.6–99.9]	99.7 [99.4–99.9]	99.8 [99.6–99.9]
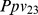	93.2 [86.7–97.6]	91.3 [81.4–97.6]	93.2 [86.6–97.6]
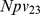	99.3 [98.9–99.7]	99.3 [98.8–99.7]	99.3 [98.9–99.7]
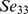	97.5 [91.0–99.9]	97.5 [91.0–99.9]	97.5 [91.0–99.9]
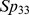	99.9 [99.7–100.0]	99.8 [99.6–100.0]	99.9 [99.7–100.0]
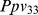	95.6 [90.2–99.1]	95.0 [87.3–99.5]	95.6 [90.2–99.1]
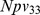	99.9 [99.7–100.0]	99.9 [99.7–100.0]	99.9 [99.7–100.0]
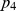	10.5 [8.6–12.5]	10.4 [8.9–11.9]	10.4 [9.0–12.0]
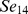	98.0 [94.4–99.8]	98.0 [94.5–99.8]	98.0 [94.5–99.7]
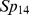	97.7 [96.6–98.6]	97.7 [96.6–98.6]	97.7 [96.6–98.6]
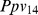	83.4 [76.3–89.4]	83.2 [76.3–89.2]	83.3 [76.4–89.3]
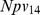	99.8 [99.3–100.0]	99.8 [99.4–100.0]	99.8 [99.4–100.0]
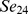	97.0 [92.9–99.4]	97.1 [93.0–99.4]	97.0 [92.9–99.4]
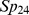	99.8 [99.6–99.9]	99.8 [99.4–100.0]	99.8 [99.6–99.9]
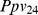	98.3 [96.7–99.4]	98.0 [94.5–99.8]	98.3 [96.7–99.4]
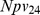	99.7 [99.2–99.9]	99.7 [99.2–99.9]	99.7 [99.2–99.9]
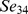	99.0 [96.2–100.0]	99.0 [96.2–100.0]	99.0 [96.2–100.0]
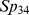	99.9 [99.7–100.0]	99.8 [99.4–100.0]	99.9 [99.7–100.0]
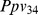	98.7 [97.2–99.8]	98.1 [94.7–99.8]	98.7 [97.2–99.7]
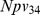	99.9 [99.6–100.0]	99.9 [99.6–100.0]	99.9 [99.6–100.0]

**Table 5:** Some information based on literature – RDT (ICT Diagnostics, Cape Town, South Africa).

Source	Local	*n*	Sensitivity(%)^1^	Specificity (%)^2^	Remarks
McMorrow et al. [4]	Kenya	4582	94.0 [93.3–94.7]	95.6 [95.0–96.1]	3
	Mozambique	2438	87.0 [85.6–88.3]	74.6 [72.8–76.3]	
	Zambia	3652	97.7 [97.2–98.1]	92.5 [91.6–93.3]	
Chinkhumba et al. [77]	Malawi	683	90.0 [82.9–94.3]	54.0 [50.1–58.2]	Febrile/ 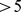 years
Kyabayinze et al. [78]	Uganda	357	98.0 [94.0–99.0]	72.0 [65.0–77.0]	Febrile/all ages
				54.0 [41.0–67.0]	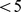 years
				78.0 [71.0–85.0]	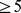 years
Portero et al. [80]	Equatorial Guinea	400	81.5 [73.8–87.8]	81.9 [76.7–86.30]	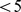 years
Moonasar et al. [81]	South Africa	405	99.5 [96.2–100.0]	96.3 [94.7–100.0]	Febrile

1,2 With 95% confidence intervals, if they are presented in source or it is possible to calculate them if they are not directly available. We use Wilson method to add CIs to the results presented by McMorrow et al. [4].

3 The sensitivity for low-density infections (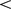
200 parasites/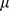
L) range from 71.7% to 100%.

4 The global sample size is 2576. Here, we consider only the patients tested by ICT rapid test.

5 To detect *P. falciparum* monoinfection the sensitivity was 69.7% [57.1–80.4] and the specificity was 73.7% [68.6–78.3].

**Table 6:** Coefficients 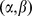 and theoretical quantiles (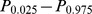) of Beta distributions for sensitivities (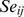) and specificities (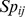): a skeptical, a optimistic and our prior beliefs distribution.

	Skeptical			Optimistic			Our Prior		
Parameters	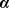	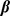	Quantiles	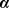	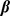	Quantiles	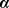	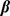	Quantiles
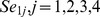	61.2	14.5	0.712–0.887	43.2	1.4	0.902–0.998	22.4	1.6	0.807–0.991
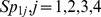	10.2	4.4	0.450–0.896	45.6	1.5	0.905–0.998	25.4	2.1	0.801–0.989
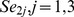	18.8	23.4	0.301–0.595	48.2	10.0	0.722–0.902	7.6	3.4	0.404–0.913
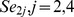	7.2	4.1	0.352–0.877	42.1	1.8	0.883–0.995	8.8	2.2	0.531–0.968
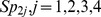	60.5	7.6	0.804–0.951	64.6	1.1	0.942–0.999	27.6	1.2	0.862–0.998
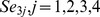	33.2	5.4	0.736–0.949	34.1	1.3	0.881–0.998	30.6	1.2	0.875–0.998
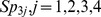	44.3	6.9	0.760–0.943	67.6	1.7	0.929–0.998	26.6	1.3	0.851–0.997

**Table 7:** Bayesian estimates of the malaria infection prevalence, sensitivities, specificities and predictive values, given by posterior means and 95% HPD intervals - Mean [95% HPD] - by age groups and fever status, using skeptical, optimistic and our prior distributions to M5.

Parameters	Skeptical 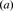	Optimistic 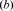	Our prior beliefs 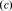	Total	Lower	DF
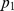	10.5 [9.0–11.9]	10.2 [8.8–11.7]	10.3 [8.8–11.7]	3834	3746	1.023
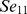	86.5 [80.5–92.5]	96.9 [93.2–99.8]	95.4 [90.3–99.5]	3747	3746	1.000
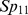	93.4 [91.0–95.8]	94.1 [92.0–96.2]	93.8 [91.6–96.0]	3760	3746	1.004
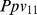	60.8 [50.9–70.4]	65.5 [56.8–74.5]	64.2 [54.9–73.4]	3710	3746	0.990
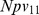	98.3 [97.5–99.1]	99.6 [99.2–100.0]	99.4 [98.8–99.9]	3917	3746	1.046
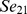	44.6 [34.4–54.9]	66.8 [57.5–75.5]	50.5 [37.7–63.2]	3824	3746	1.021
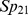	99.6 [99.4–99.8]	99.8 [99.7–99.9]	99.8 [99.6–99.9]	3933	3746	1.050
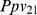	93.0 [88.7–96.9]	97.5 [95.4–99.3]	96.6 [93.7–99.1]	3747	3746	1.000
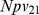	93.9 [92.4–95.3]	96.3 [95.2–97.5]	94.6 [93.1–96.2]	3768	3746	1.006
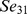	89.5 [81.6–96.2]	96.4 [91.9–99.9]	96.0 [90.7–99.9]	3714	3746	0.991
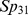	99.7 [99.5–99.9]	99.8 [99.6–99.9]	99.8 [99.7–100.0]	4068	3746	1.086
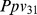	97.0 [94.9–98.8]	98.2 [96.7–99.5]	98.5 [97.1–99.8]	3650	3746	0.974
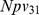	98.8 [97.8–99.6]	99.6 [99.1–100.0]	99.5 [98.9–100.0]	3665	3746	0.978
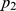	10.5 [9.0–11.9]	10.2 [8.8–11.7]	10.3 [8.8–11.7]	3834	3746	1.023
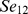	84.3 [77.1–90.8]	96.5 [92.1–99.8]	94.1 [87.5–99.4]	3778	3746	1.009
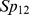	96.4 [93.9–98.7]	97.9 [96.1–99.4]	97.5 [95.5–99.3]	3809	3746	1.017
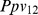	73.8 [60.2–87.0]	84.3 [73.5–94.9]	81.5 [69.6–93.1]	3802	3746	1.015
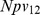	98.1 [97.2–98.9]	99.6 [99.1–100.0]	99.3 [98.5–99.9]	3863	3746	1.031
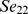	82.0 [69.1–93.2]	94.3 [88.7–98.9]	87.6 [76.7–97.0]	3688	3746	0.985
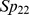	99.6 [99.4–99.8]	99.8 [99.7–99.9]	99.8 [99.6–99.9]	3933	3746	1.050
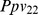	96.1 [93.7–98.2]	98.2 [96.8–99.5]	98.0 [96.4–99.5]	3747	3746	1.000
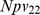	97.9 [96.5–99.2]	99.4 [98.7–99.9]	98.6 [97.4–99.7]	3729	3746	0.995
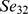	89.8 [82.6–96.8]	96.2 [91.3–99.8]	96.1 [91.0–99.8]	3716	3746	0.992
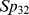	99.7 [99.5–99.9]	99.8 [99.6–99.9]	99.8 [99.7–100.0]	4068	3746	1.086
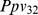	97.0 [94.9–98.8]	98.2 [96.7–99.5]	98.5 [97.1–99.7]	3758	3746	1.003
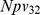	98.8 [98.0–99.6]	99.6 [99.0–100.0]	99.6 [99.0–100.0]	3910	3746	1.044
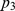	3.3 [2.4–4.2]	3.2 [2.4–4.1]	3.2 [2.3–4.1]	3738	3746	0.998
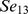	87.0 [81.0–92.5]	97.3 [93.9–99.9]	96.0 [91.5–99.7]	3768	3746	1.006
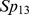	98.6 [98.0–99.1]	98.7 [98.1–99.2]	98.7 [98.1–99.2]	3782	3746	1.010
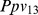	67.6 [56.0–78.5]	71.6 [61.3–81.8]	70.6 [59.8–80.8]	3665	3746	0.978
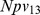	99.6 [99.3–99.8]	99.9 [99.8–100.0]	99.9 [99.7–100.0]	3914	3746	1.045
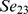	63.7 [53.7–73.4]	82.2 [75.3–89.4]	79.1 [68.7–88.9]	3793	3746	1.013
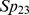	99.6 [99.4–99.8]	99.8 [99.7–99.9]	99.8 [99.6–99.9]	3933	3746	1.050
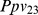	84.7 [75.9–92.5]	93.3 [87.8–98.0]	92.8 [87.0–97.8]	3732	3746	0.996
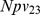	98.8 [98.3–99.2]	99.4 [99.1–99.7]	99.3 [98.9–99.7]	3956	3746	1.056
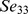	92.4 [86.2–97.8]	98.3 [95.3–100.0]	98.3 [95.3–100.0]	3725	3746	0.994
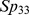	99.7 [99.5–99.9]	99.8 [99.6–99.9]	99.8 [99.7–100.0]	4068	3746	1.086
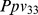	90.7 [84.4–96.2]	94.2 [89.3–98.5]	95.2 [90.5–99.2]	3771	3746	1.007
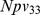	99.7 [99.5–99.9]	99.9 [99.8–100.0]	99.9 [99.8–100.0]	3889	3746	1.038
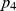	10.5 [9.0–11.9]	10.2 [8.8–11.7]	10.3 [8.8–11.7]	3834	3746	1.023
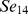	91.1 [86.7–95.0]	98.3 [96.2–99.9]	97.9 [95.3–99.8]	3813	3746	1.018
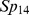	97.4 [96.3–98.3]	97.8 [96.8–98.6]	97.7 [96.6–98.6]	3828	3746	1.022
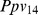	80.2 [73.1–86.7]	83.4 [77.2–89.6]	82.7 [76.2–89.0]	3740	3746	0.998
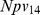	98.9 [98.4–99.4]	99.8 [99.6–100.0]	99.8 [99.5–100.0]	3956	3746	1.056
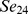	94.3 [90.0–98.3]	97.4 [94.7–99.6]	96.2 [92.6–99.2]	3706	3746	0.989
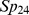	99.6 [99.4–99.8]	99.8 [99.7–99.9]	99.8 [99.6–99.9]	3933	3746	1.050
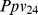	96.6 [94.6–98.4]	98.3 [96.9–99.5]	98.2 [96.7–99.5]	3807	3746	1.016
	99.3 [98.8–99.8]	99.7 [99.4–100.0]	99.6 [99.2–99.9]	3833	3746	1.023
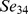	95.9 [92.6–98.9]	99.0 [97.4–100.0]	99.1 [97.4–100.0]	3802	3746	1.015
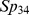	99.7 [99.5–99.9]	99.8 [99.6–99.9]	99.8 [99.7–100.0]	4068	3746	1.086
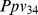	97.2 [95.3–98.9]	98.3 [96.8–99.5]	98.6 [97.2–99.8]	3743	3746	0.999
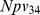	99.5 [99.1–99.9]	99.9 [99.7–100.0]	99.9 [99.7–100.0]	3896	3746	1.040

Raftery and Lewis convergence diagnostics related with our prior distribution and some measures of model selection (in the footnote).

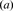
DIC: 168.824, AIC: 200.567, BIC: 322.703, and p-value: 0.007.

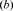
DIC: 121.197, AIC: 149.205, BIC: 271.341, and p-value: 0.485.


DIC: 115.934, AIC: 143.614, BIC: 265.750, and p-value: 0.768.

## Results and Discussion

### Results with Non-informative Priors

The hypothesis of conditional independence was checked using LORC, correlation residual plots and bivariate residuals and biplots. No statistical evidence of local dependence was detected, however, taking into account that in certain situations some of these tools may not detect the HCI [36], multinomial fixed effects models with conditional dependence modeled by covariances between tests within classes (e.g. [39], [62]) were also explored following biological reasons. The fitting of such models (based on DIC and *p*-value and predictive frequencies of each pattern) did not show relevant information compared with simpler models. Even if these models offer a closer description of reality, the balance with parsimony remains an important issue. Interpretability and identifiability problems may arise from models with a larger number of parameters. Moreover, it is well-known that models assuming different dependency structures can provide different parameter estimates and lead to very different interpretations, in spite of their similarity in terms of adjustment measures [37]. In addition, in a Bayesian context, it might be difficult to elicit prior distributions to the covariances or random effects coefficients [20].

Following the HCI exhaustive validation and analysis of the other described topics, we gave a special attention to the results of two mentioned models - M1 and M2 - and three related models with constraints: M3, M4 and M5. Briefly,

#### M1

The typical model (with constraints) admits a different prevalence for each subpopulation and the sensitivities and specificities of each test are the same across subpopulations (i.e., for 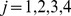, 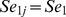, 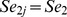, 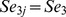, 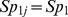, 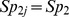, and 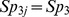),

#### M2

The general model (without constraints) that assumes possible differences across subpopulations in terms of prevalence, sensitivities and specificities of each test,

#### M3

This model with constraints admits a different prevalence across subpopulations, the specificity of microscopy is equal across subpopulations and also the specificity of PCR (i.e., for 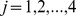, 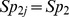 and 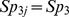). All the remaining parameters vary across subpopulations,

#### M4

The general model - M2 - adding: 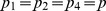,

#### M5

The same constraints of the M3 adding: 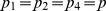.

In a first step, we explored our malaria dataset with non-informative prior distributions for all parameters related with test characteristics (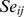 and 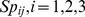 and 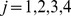), using Beta(1,1) distributions, equivalent to Uniform distributions over the interval [0,1]. For the prevalence in the four subpopulations (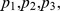 and 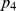), the Uniform distribution was considered – U(0,0.5). For the five selected models (M1, M2, M3, M4, and M5), no convergence problems were found and some of the measures that we have been discussing are presented in Table 3.

The assumption of constant test accuracy across subpopulations with different malaria infection prevalence was evaluated though model M1 and it seems to reveal a poorer fit. M2 admits differences across subpopulations in terms of prevalence, sensitivities and specificities of each test and compared with the model M1 seems to fit better. M4 adds only the possibility of febrile and afebrile under-five children and febrile with at least five years old having similar prevalence, but the test’ characteristics varying across subpopulations. This model presents a DIC similar to M2. M3 and M5 present yet better DICs. However, M3 presents a DIC not substantially different from M4. Between M3 and M5, the difference in DICs is also less than 5. Following the recommendations of the BUGS Project [68], we present the estimated parameters according to the three models to investigate possible discrepancies in estimates given by these models (see Table 4).

The posterior inferences, which combine prior information (or lack of it) with data information via Bayes’ theorem, are summarized in Table 4, presenting the posterior means and 95% credibility interval. Additional to the original parameters of the models, the positive predictive values (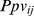) and negative predictive values (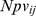) were also indirectly estimated using their relationship with the prevalence, sensitivities and specificities (see expressions, for example, in [29]). M3 and M5 produce similar results. M4 presents some discrepancies at least in some predictive values. According to the parsimony principle, M5 is the simplest model and all criteria of selection and goodness-of-fit are satisfactory, consequently, it is elected as the final model to fit the malaria dataset. Further analysis will be needed to see how the inferences change with different types of informative priors.

### Results with Informative Priors

Some information was collected in published works to help us in the choice of the prior distributions for each parameter of our elected model. An accurate estimation is not necessary, this process is flexible and seeks some general knowledge. Additionally, expert opinions were considered in final informative prior distributions.

#### RDT

Table 5 shows a range of values for sensitivities and specificities of the RDT test (ICT Diagnostics, Cape Town, South Africa), according to local area and age groups or fever status. Bendezu et al. [74] describe that the same RDT used in different places showed different results (probably related to different conditions like temperature, humidity, characteristics of the malaria parasites, etc.). The study design, the sample size and statistical analysis of each study also contribute to different findings across different studies.

#### Microscopy

As microscopy is usually the reference test, very few papers present its sensitivity and specificity. Speybroeck et al. [14], using a Bayesian approach, found the following posterior means and 95% credibility intervals for sensitivity by survey: 53.0% ([42.0–70.0] in Vietnam, 90.0% [72.0–100.0] in Peru Iquitos, 89.0% [71.0–100.0] in Peru Jaen, Cambodia - Survey 1, and Cambodia - Survey 2. In terms of specificities the lower bounds of credibility intervals were higher than 94%. These authors give details about their prior distributions (Uniform) based on expert opinion. Through the classical analysis, using the PCR as a reference test, Batwala et al. [75] explored the performance of microscopy as a function of laboratory experience – health centre (HC) microscopy and expert microscopy. The point estimates and the 95% CI are reported in both cases. In patients with 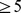 years, the specificity of HC microscopy was 95.7% [90.8–98.4] and the expert microscopy was 98.6% [94.9–99.8]. In children under-five, the specificities of HC microscopy and the expert microscopy were 89.0% [79.5–95.1] and 94.5% [86.6–98.5], respectively. The overall sensitivity of HC microscopy was 47.2% (36.5–58.1) and the sensitivity of expert microscopy was 46.1% [35.4–57.0].

#### PCR

The Bayesian analysis performed by Speybroeck et al. [14], in the absence of a reference test, highlighted that PCR is more sensitive than microscopy and the estimates for sensitivity vary from 95.0% [89.0–100.0] in Vietnam to 98.0% [95.0–100.0] in Peru Iquitos and Peru Jaen. In terms of specificity the results are the following: Vietnam –97.0% [95.0–100.0], Peru Iquitos –99.0% [98.0–100.0] and Peru Jean –100.0% (99.0–100.0). Coleman et al. [76] studied the performance of PCR at different parasite densities relative to expert laboratory microscopy, for active surveillance of *Plasmodium falciparum* and *Plasmodium vivax*, and reported that PCR was sensitive 95.7% [84.3–99.3] and specific 98.1% [97.8–98.4] for malaria at parasite densities 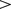
500/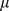
l. However, the sensitivity of PCR dropped off markedly for parasite densities 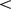
500/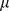
l. The specificity was constantly high, with a minimum lower bound of the CI equal to 97.4%.

Based on expert opinions on malaria diagnosis in STP and published works focusing on similar diagnostic tests, we consider Beta distributions to represent a pessimistic or skeptical, a optimistic and our prior beliefs distribution. The theoretical parameters and quantiles of these distributions are presented in Table 6. Our prior distributions for each subpopulation express: (i) In general, the RDT test (ICT Diagnostics) presents a similar behavior in each subpopulation; (ii) PCR is usually more sensitive than microscopy; (iii) Microscopy is usually more specific than PCR; (iv) Sensitivity of microscopy is slightly better in the febrile individuals. Except for the specificity of microscopy, we consider the same prior distribution for each parameters across the four subpopulations (see Table 6), even though M5 only admits that the specificity of microscopy and the specificity of PCR are equal across subpopulations.

In Table 7, we present again the the posterior means and HPD intervals for each parameters through model M5 with a skeptical, a optimistic and our prior beliefs distributions. We check the convergence of all parameters, and not just those of interest, before proceeding to make any inference, using the trace plots and Gelman-Rubin and Raftery-Lewis convergence diagnostics measures (the last one is presented in Table 7). DIC, AIC, BIC and Bayesian *p*-values are also indicated in Table 7. These measures favors our prior beliefs distribution, but the more optimistic prior is yet admissible. The Bayesian *p*-value (0.007) associated to model M5 with a skeptical prior distribution reveals a prior-data conflict. Compared with the results obtained using M5 with non-informative priors, it can be seen (last columns in Table 4) that our prior information contributes to an increase of the sensitivities of microscopy and PCR, in afebrile children under-five. In the febrile children under-five, the sensitivities of RDT and PCR are also improved. The rest of the parameters are quite similar. In afebrile children under-five, the sensitivity of microscopy (even under an optimistic prior) is very low. This finding is not unexpected since other previous studies have reported low values, when this test is not considered as a gold standard, pointing out that asymptomatic cases often have undetectable malaria parasites by microscopy [14], [75].

Our study is associated to a small region composed of two islands, where an intensive malaria control programme aimed at pre-elimination of malaria was developed with success, where prevalences were highly reduced in general and many positive cases had no malaria clinical signs associated. Therefore, the data and results may not be comparable to other regions elsewhere, with higher prevelances obtained by microscopy or RDT. As mentioned before different results may reflect different factors and the word “comparison” may be too strong. Nevertheless, Chinkhumba et al. [77] state that malaria RDTs must have both sensitivity and specificity above 95% in field setting. These authors report that the sensitivity of the RDTs evaluated in their study are similar to the results of other published studies. However, they found a low specificity in febrile patients above 5 years of age. Kyabayinze et al. [78] alert to the low specificity of the ICT rapid test especially in children below 5 years of age. In our study, in terms of the RDT test, for the afebrile children under-five, the specificity estimated by posterior mean is 93.8% and in the remaining subpopulations is above 97.5%. In terms of sensitivity, for febrile children under-five, we find: 94.1% [87.5–99.4]. In all subpopulations, the positive predictive values of RDT are lower than other tests. The PCR yields reliable results in four subpopulations.

### Comparison with Other Approaches

The sensitivity and specificity of several rapid malaria diagnostic tests have been estimated using the microscopy as a gold standard. However, the previous measures may change substantially considering the polymerase chain reaction (e.g. [6]) as reference. Only with the purpose of understanding the implications of the classical statistical approach (which is still common in medical literature), in this subsection, we present the performance measures of RDT, admitting the microscopy as a gold standard (Table 8). The use of interval estimation for reporting performance measures is recommended but the Wald method may not be appropriate. Thus, the Clopper-Pearson or exact binomial, Wilson (or score), Agresti-Coull and Jeffreys methods are also calculated to obtain confidence intervals (see Table 8).

**Table 8:** Point estimates and 95% confidence intervals, though five different methods, for the sensitivity and the specificity of RDT in each subpopulation 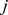 (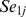 and 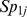) and overall (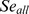 and 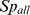), using microscopy as a gold standard.

Parameter	Point estimate 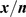 (%)	Wald	Clopper-Pearson	Wilson	Agresti-Coull	Jeffreys
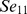	21/23 (91.3)	79.8–100.0	72.0–98.9	73.2–97.6	72.0–98.8	74.9–98.1
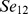	23/24 (95.8)	87.8–100.0	78.9–99.9	79.8–99.3	78.1–100.0	82.1–99.5
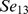	40/44 (90.9)	82.4–99.4	78.3–97.5	78.8–96.4	78.3–97.0	79.8–96.9
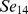	98/100(98.0)	95.3–100.0	93.0–99.8	93.0–99.4	92.6–99.9	93.7–99.6
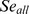	182/191 (95.3)	92.3–98.3	91.2–97.8	91.3–97.5	91.2–97.6	91.6–97.6
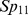	420/472 (89.0)	86.2–91.8	85.8–91.7	85.8–91.5	85.8–91.5	85.9–91.6
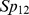	211/217 (97.2)	95.1–99.4	94.1–99.0	94.1–98.7	94.0–98.9	94.4–98.8
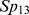	1537/1565 (98.2)	97.6–98.9	97.4–98.8	97.4–98.8	97.4–98.8	97.5–98.8
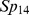	851/872 (97.6)	96.6–98.6	96.3–98.5	96.3–98.4	96.3–98.4	96.4–98.5
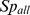	3019/3126 (96.6)	95.9–97.2	95.9–97.2	95.9–97.2	95.9–97.2	95.9–97.2

The sensitivities are estimated based on smaller denominators and the corresponding Wald interval tends to provide higher lower bounds for the 95% CI than the other recommended methods. The specificities in all subpopulations are estimated from larger sample sizes and the five methods give similar results. The Wald method is not appropriate to report the performance of a diagnostic test, in particular, when the prevalence of an infection is small (high) because erratic values for the sensitivity (specificity) may occur.

Using the classical analysis (see Table 8), it is emphasized that the sensitivities of RDT are lower than the Bayesian estimates in three subpopulations. The exception is the afebrile with less than five years old. The 95% HPD intervals are narrower than 95% confidence intervals. Paradoxically, in our application, the Wald confidence intervals are the ones that resemble more the Bayesian credibility intervals, particulary for the sensitivities. Nevertheless, this method is not recommended for the typical values of sensitivities and specificities. One reviewer suggested a simple ad-hoc method, assessing the sensitivity of a method as the percentage of positive responses in the group that has positive values for both other tests and specificity as the percentage of negative among those that have negative values for both other tests. There is some proximity between this approach and the composite reference standards proposed by Alonzo and Pepe [79]. Particularly for the sensitivities, as the sample size decreases because the discrepant results between the two reference tests are discarded, the 95% confidence intervals are wider (data not shown). However, for RDT test, combining the PCR and microscopy results, the point estimates are closer to posterior means obtained by Bayesian analysis. In terms of specificities, the reduction of the sample size has less effect because the samples are already larger, leading to smoother differences between the three approaches.

In addition to the philosophical perspective that prior information is an important source to characterize a problem in a more realistic way, in this application, the major advantage of the Bayesian approach is that the subpopulations parameters are estimated by narrower intervals, compared with other approaches. Analyzing the four populations at once and informative priors could prevent identifiability problems. Furthermore, the use of constraints helps enhance the modeling versatility because it is possible to explore the differences and similarities between subpopulations.

### Conclusions

The accuracy of diagnostic tests for the malaria diagnosis based on the optical microscopy as a gold standard has been criticized and alternative statistical approaches have emerged without wrongly assuming any of the diagnostic tests as a perfect gold standard. Some studies have reported the performance measures in different populations, exhibiting some differences. Here, we have addressed this problem with a novel Bayesian approach, in the malaria context, which avoids defining a gold standard and provides estimates to the malaria infection prevalence and performance measures in different subpopulations simultaneously. Some deterministic and probabilistic constraints were considered to express some available knowledge or suppositions of experts and published literature about laboratory diagnosis of malaria.

Different models were explored, some of them providing similar results. The elected model was the one that considers a different prevalence in the afebrile individuals with at least five years old and the remaining three groups with the same prevalence. This model admitted the specificity of microscopy and the specificity of PCR are equal across subpopulations and their sensitivities are different. In terms of the performance measures of RDT no constraints are imposed in each subpopulations.

The data information collected in STP seems to be dominant, since the main findings were quite stable when we use different prior distributions. When we consider a positive expectation, using an optimistic prior, or a more skeptical position (pessimistic prior), yielded the same results in terms of the order in which the tests were arranged and even in terms of the magnitude of some performance measures.

In the afebrile individuals with at least five years old, the posterior estimate of the malaria infection prevalence was around 3.2% [2.3–4.1] and in the remaining studies groups around 10.3% [8.8–11.7]. Microscopy had poor sensitivity compared to the other tests, particularly, in afebrile children under-five years old 50.5% [37.7–63.2]. The PCR yielded reliable results in four subpopulations. However, in resource-limited settings, the PCR is not yet accepted as a primary diagnostic test in malaria diagnosis. According to Chinkhumba et al. [77], malaria RDTs must have both sensitivity and specificity above 95% in field setting. In STP the results seems to satisfy this conditions in adults and children with at least five years old. In children under-five, the sensitivity was lower than this target. In all subpopulations, data provide enough evidence to suggest caution with the positive predictive values of the RDT.

## Supporting Information

Appendix S1 **An example of the code corresponding to model M5.**
(PDF)
