# Supplementary material for: Bayesian Latent Class Models in Malaria Diagnosis
Source: PLoS One. 2012 Jul 23;7(7):e40633. doi: 10.1371/journal.pone.0040633 (PMC3402519; doi:10.1371/journal.pone.0040633)
Supplement: Appendix S1 — An example of the code corresponding to model M5. (PDF) [file pone.0040633.s001.pdf]

Appendix S1 - An example of the code corresponding to model M5

```
model;
{
x[1:Q1, 1:Q2] ~ dmulti(p1[1:Q1, 1:Q2], n1) # Age < 5 years; Without fever
y[1:Q1, 1:Q2] ~ dmulti(p2[1:Q1, 1:Q2], n2) # Age < 5 years; With fever
z[1:Q1, 1:Q2] ~ dmulti(p3[1:Q1, 1:Q2], n3) # Age >= 5 years; Without fever
w[1:Q1, 1:Q2] ~ dmulti(p4[1:Q1, 1:Q2], n4) # Age >= 5 years; With fever

#Seij and Spij - sens. and spec. test i=1,2,3 (RDT, microscopy, PCR, respectively) in jth population
#pij: prevalence in the jth population

# Restriction pi1=pi2=pi4
pi2<-pi1
pi4<-pi1

# Restrictions Sp21=Sp22=Sp23=Sp24; Sp31=Sp32=Sp33=Sp34
Sp22<-Sp21
Sp23<-Sp21
Sp24<-Sp21
Sp32<-Sp31
Sp33<-Sp31
Sp34<-Sp31

p1[1,1] <- pi1*Se11*Se21*Se31 + (1-pi1)*(1-Sp11) *(1-Sp21) *(1-Sp31)  #pattern (111)
p1[1,2] <- pi1*Se11*Se21*(1-Se31) + (1-pi1)*(1-Sp11) *(1-Sp21) *Sp31  # (110)
p1[1,3] <- pi1*Se11*(1-Se21)*Se31 + (1-pi1)*(1-Sp11) *Sp21*(1-Sp31)   # (101)
p1[1,4] <- pi1*Se11*(1-Se21)*(1-Se31) + (1-pi1)*(1-Sp11) *Sp21 *Sp31  # (100)
p1[2,1] <- pi1*(1-Se11)*Se21*Se31 + (1-pi1)*Sp11*(1-Sp21) *(1-Sp31)   # (011)
p1[2,2] <- pi1*(1-Se11)*Se21*(1-Se31) + (1-pi1)*Sp11 *(1-Sp21) *Sp31  # (010)
p1[2,3] <- pi1*(1-Se11)*(1-Se21)*Se31 + (1-pi1)*Sp11*Sp21*(1-Sp31)    # (001)
p1[2,4] <- pi1*(1-Se11)*(1-Se21)*(1-Se31) + (1-pi1)*Sp11*Sp21*Sp31    # (000)

p2[1,1] <- pi2*Se12*Se22*Se32 + (1-pi2)*(1-Sp12) *(1-Sp22) *(1-Sp32)
p2[1,2] <- pi2*Se12*Se22*(1-Se32) + (1-pi2)*(1-Sp12) *(1-Sp22) *Sp32
```

```
p2[1,3] <- pi2*Se12*(1-Se22)*Se32 + (1-pi2)*(1-Sp12) *Sp22*(1-Sp32)
p2[1,4] <- pi2*Se12*(1-Se22)*(1-Se32) + (1-pi2)*(1-Sp12) *Sp22 *Sp32
p2[2,1] <- pi2*(1-Se12)*Se22*Se32 + (1-pi2)*Sp12*(1-Sp22) *(1-Sp32)
p2[2,2] <- pi2*(1-Se12)*Se22*(1-Se32) + (1-pi2)*Sp12 *(1-Sp22) *Sp32
p2[2,3] <- pi2*(1-Se12)*(1-Se22)*Se32 + (1-pi2)*Sp12*Sp22*(1-Sp32)
p2[2,4] <- pi2*(1-Se12)*(1-Se22)*(1-Se32) + (1-pi2)*Sp12*Sp22*Sp32

p3[1,1] <- pi3*Se13*Se23*Se33 + (1-pi3)*(1-Sp13) *(1-Sp23) *(1-Sp33)
p3[1,2] <- pi3*Se13*Se23*(1-Se33) + (1-pi3)*(1-Sp13) *(1-Sp23) *Sp33
p3[1,3] <- pi3*Se13*(1-Se23)*Se33 + (1-pi3)*(1-Sp13) *Sp23*(1-Sp33)
p3[1,4] <- pi3*Se13*(1-Se23)*(1-Se33) + (1-pi3)*(1-Sp13) *Sp23 *Sp33
p3[2,1] <- pi3*(1-Se13)*Se23*Se33 + (1-pi3)*Sp13*(1-Sp23) *(1-Sp33)
p3[2,2] <- pi3*(1-Se13)*Se23*(1-Se33) + (1-pi3)*Sp13 *(1-Sp23) *Sp33
p3[2,3] <- pi3*(1-Se13)*(1-Se23)*Se33 + (1-pi3)*Sp13*Sp23*(1-Sp33)
p3[2,4] <- pi3*(1-Se13)*(1-Se23)*(1-Se33) + (1-pi3)*Sp13*Sp23*Sp33

p4[1,1] <- pi4*Se14*Se24*Se34 + (1-pi4)*(1-Sp14) *(1-Sp24) *(1-Sp34)
p4[1,2] <- pi4*Se14*Se24*(1-Se34) + (1-pi4)*(1-Sp14) *(1-Sp24) *Sp34
p4[1,3] <- pi4*Se14*(1-Se24)*Se34 + (1-pi4)*(1-Sp14) *Sp24*(1-Sp34)
p4[1,4] <- pi4*Se14*(1-Se24)*(1-Se34) + (1-pi4)*(1-Sp14) *Sp24 *Sp34
p4[2,1] <- pi4*(1-Se14)*Se24*Se34 + (1-pi4)*Sp14*(1-Sp24) *(1-Sp34)
p4[2,2] <- pi4*(1-Se14)*Se24*(1-Se34) + (1-pi4)*Sp14 *(1-Sp24) *Sp34
p4[2,3] <- pi4*(1-Se14)*(1-Se24)*Se34 + (1-pi4)*Sp14*Sp24*(1-Sp34)
p4[2,4] <- pi4*(1-Se14)*(1-Se24)*(1-Se34) + (1-pi4)*Sp14*Sp24*Sp34

# PPV and NPV

# Test RDT
ppv11<-pi1*Se11/(pi1*Se11+(1-pi1)*(1-Sp11))
ppv12<-pi2*Se12/(pi2*Se12+(1-pi2)*(1-Sp12))
ppv13<-pi3*Se13/(pi3*Se13+(1-pi3)*(1-Sp13))
ppv14<-pi4*Se14/(pi4*Se14+(1-pi4)*(1-Sp14))

npv11<-(1-pi1)*Sp11/((1-pi1)*Sp11+pi1*(1-Se11))
npv12<-(1-pi2)*Sp12/((1-pi2)*Sp12+pi2*(1-Se12))
npv13<-(1-pi3)*Sp13/((1-pi3)*Sp13+pi3*(1-Se13))
npv14<-(1-pi4)*Sp14/((1-pi4)*Sp14+pi4*(1-Se14))
```

```
# Test Microscopy
ppv21<-pi1*Se21/(pi1*Se21+(1-pi1)*(1-Sp21))
ppv22<-pi2*Se22/(pi2*Se22+(1-pi2)*(1-Sp22))
ppv23<-pi3*Se23/(pi3*Se23+(1-pi3)*(1-Sp23))
ppv24<-pi4*Se24/(pi4*Se24+(1-pi4)*(1-Sp24))

npv21<-(1-pi1)*Sp21/((1-pi1)*Sp21+pi1*(1-Se21))
npv22<-(1-pi2)*Sp22/((1-pi2)*Sp22+pi2*(1-Se22))
npv23<-(1-pi3)*Sp23/((1-pi3)*Sp23+pi3*(1-Se23))
npv24<-(1-pi4)*Sp24/((1-pi4)*Sp24+pi4*(1-Se24))

# Test PCR
ppv31<-pi1*Se31/(pi1*Se31+(1-pi1)*(1-Sp31))
ppv32<-pi2*Se32/(pi2*Se32+(1-pi2)*(1-Sp32))
ppv33<-pi3*Se33/(pi3*Se33+(1-pi3)*(1-Sp33))
ppv34<-pi4*Se34/(pi4*Se34+(1-pi4)*(1-Sp34))

npv31<-(1-pi1)*Sp31/((1-pi1)*Sp31+pi1*(1-Se31))
npv32<-(1-pi2)*Sp32/((1-pi2)*Sp32+pi2*(1-Se32))
npv33<-(1-pi3)*Sp33/((1-pi3)*Sp33+pi3*(1-Se33))
npv34<-(1-pi4)*Sp34/((1-pi4)*Sp34+pi4*(1-Se34))

# Bayesian p-value, according to Pascale Nrette, 2008

##  "Test statistic" determined for observed data
for (i in 1:Q1) {
        for (j in 1:Q2)   {
        tx[i,j]<-pow((x[i,j]-p1[i,j]*n1),2)/(p1[i,j]*n1)
        ty[i,j]<-pow((y[i,j]-p2[i,j]*n2),2)/(p2[i,j]*n2)
        tz[i,j]<-pow((z[i,j]-p3[i,j]*n3),2)/(p3[i,j]*n3)
        tw[i,j]<-pow((w[i,j]-p4[i,j]*n4),2)/(p4[i,j]*n4)
        }
}

##  Prediction
xpred [1:Q1, 1:Q2] ~ dmulti(p1[1:Q1, 1:Q2], 495)
ypred [1:Q1, 1:Q2] ~ dmulti(p2[1:Q1, 1:Q2], 241)
zpred [1:Q1, 1:Q2] ~ dmulti(p3[1:Q1, 1:Q2], 1609)
```

```
wpred [1:Q1, 1:Q2] ~ dmulti(p4[1:Q1, 1:Q2], 972)

# "Test statistic" determined for predicted data
for(i in 1:Q1)
{
        for(j in 1:Q2)
        {
        txpred[i,j]<-pow((xpred[i,j]-p1[i,j]*n1),2)/(p1[i,j]*n1)
        typred[i,j]<-pow((ypred[i,j]-p2[i,j]*n2),2)/(p2[i,j]*n2)
        tzpred[i,j]<-pow((zpred[i,j]-p3[i,j]*n3),2)/(p3[i,j]*n3)
        twpred[i,j]<-pow((wpred[i,j]-p4[i,j]*n4),2)/(p4[i,j]*n4)
        }
}

## Bayesian p-value
ttotal<-sum(tx[,])+sum(ty[,])+sum(tz[,])+sum(tw[,])
ttotalpred<-sum(txpred[,])+sum(typred[,])+sum(tzpred[,])+sum(twpred[,])

bayespvalue<-step(ttotalpred-ttotal)
}
```
